# Emerging Therapeutic Approaches for Tic Alleviation in Tourette Syndrome: The Role of Micronutrients

**DOI:** 10.3390/neurolint18010007

**Published:** 2025-12-26

**Authors:** Samskruthi Madireddy, Sahithi Madireddy

**Affiliations:** 1 Department of Neuroscience, Johns Hopkins University, Baltimore, MD 21218, USA; 2 Department of Neurology, Johns Hopkins University School of Medicine, Baltimore, MD 21205, USA

**Keywords:** Tourette’s syndrome, tic disorder, micronutrients, vitamin D, vitamin B6, vitamin A, iron, magnesium, zinc, copper

## Abstract

Tourette syndrome (TS), or Tourette’s, is a tic disorder (TD) belonging to a group of neuropsychiatric conditions marked by recurrent motor movements or vocalizations known as tics. TD, including TS, typically begins in childhood between 4 and 18 years of age and affects approximately 3% of children and adolescents. The etiology and pathogenesis of TD are multifactorial, involving genetic, immunologic, psychological, and environmental factors. Evidence suggests that neurotransmitter dysregulation, particularly within the cortical dopaminergic networks of the basal ganglia and limbic system, which support motor control and cognition, may be involved in the development of TD. Nutritional factors may modulate TD through various mechanisms, including effects on neurotransmitter synthesis and metabolism, neurodevelopment, neural architecture, and neuroimmune activity. This review integrates current evidence on the roles of vitamins D, B6, and A, as well as iron, magnesium, zinc, and copper, in TD. For each micronutrient, its physiological and neurobiological functions are discussed, along with possible mechanistic links to TD pathophysiology. Additionally, we summarize the impact of nutrient deficiencies and assess available evidence regarding their potential therapeutic potential role in TD management. Overall, this synthesis highlights how nutritional status may influence TD onset and symptom severity, suggesting that nutrient-based interventions could potentially serve as valuable adjunctive strategies in treatment.

## 1. Introduction

Tic disorders (TDs) are common neurodevelopmental conditions characterized by the occurrence of tics, which are brief, repetitive motor movements or vocalizations often preceded by a premonitory urge [1,2,3,4,5,6,7]. Tics show considerable variability both between individuals and within the same individual over time, including differences in intensity, complexity, location, and frequency [4]. TDs can be further classified based on clinical course and symptom presentation. The main subtypes include transient tic disorder (TTD), chronic motor or vocal tic disorder (CTD), and Tourette syndrome (TS) [8,9]. The prevalence of TD varies, with TS being less common than other persistent tic disorders. A CDC report estimated that approximately 0.3% of children aged 3 to 17 years in the United States, about 174,000 between 2016 and 2019, were diagnosed with TS [10,11]. TS is also frequently associated with comorbid neuropsychiatric conditions. Over two-thirds of affected individuals experience co-occurring disorders such as obsessive–compulsive disorder (OCD), autism spectrum disorder (ASD), attention-deficit/hyperactivity disorder (ADHD), sleep disturbances, depression, and anxiety [12,13,14,15].

The etiology and pathogenesis of TD remain incompletely understood, but multiple interacting factors are contributing, including genetic, microbiological, immunologic, psychological, and environmental components [16,17,18]. A widely supported hypothesis proposes disinhibition within the cortico-striato-thalamo-cortical (CSTC) circuit, a neural loop central to motor control and cognitive regulation [19]. Such disinhibition may be partly caused by excessive dopaminergic activity in the striatum, arising from increased dopamine (DA) release or enhanced postsynaptic DA receptor sensitivity [19,20]. This concept aligns with extensive evidence linking dopaminergic imbalance in cortico-basal ganglia pathways to the pathophysiology of CTD [21,22]. Neuroimaging studies have shown increased DA transporter binding in individuals with TD, while immunologic investigations have detected anti-dopamine D2 receptor autoantibodies correlated with symptom severity [23]. Some researchers further suggest that spontaneous fluctuations in tic manifestation may reflect transient alterations in dopaminergic signaling [22]. In addition to DA, other neurotransmitter systems have been implicated in TD pathology, including glutamate, γ-aminobutyric acid (GABA), serotonin, norepinephrine, acetylcholine, and histamine [24,25,26,27].

Growing evidence and anecdotal reports suggest that environmental influences, particularly diet and nutrition, may contribute to tic symptomatology [28]. Nutritional factors can influence the dopaminergic system by altering neurotransmitter synthesis and metabolism, modulating gut microbiota composition, and driving neuroinflammatory processes, highlighting a complex interplay relevant to TD [29]. Although the importance of nutrition has been established in various neurological and psychiatric conditions, its specific relationship with TS and other TD remains underexplored. Evidence shows that adjuvant treatment including micronutrients may have therapeutic benefits against neurological disorders [30]. This review summarizes current evidence on the potential involvement of key nutrients in TD, including vitamin D, vitamin B6, vitamin A, iron, magnesium, zinc, and copper. By consolidating findings, it aims to advance understanding of TD pathophysiology and explore the potential of targeted nutritional strategies in its management and prevention.

## 2. Vitamin D (Vit D)

**Figure 1:**
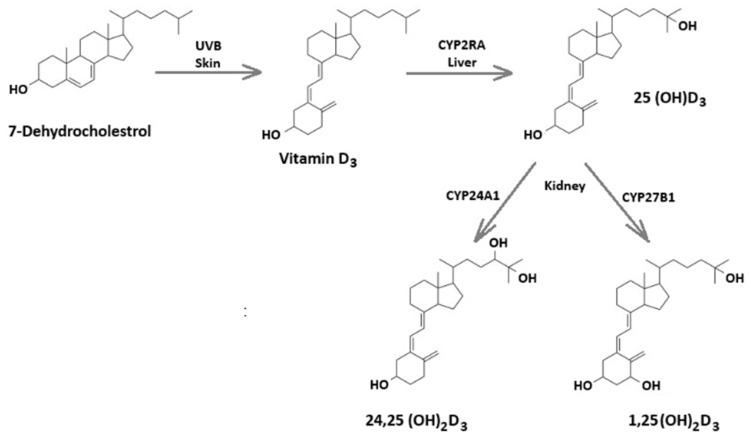
Overview of vitamin D metabolic enzymes and sites of metabolism.

Vitamin D is a neuroactive steroid hormone essential for skeletal health, immune regulation, and neural function [31,32]. Its biologically active precursor, vitamin D_3_ (cholecalciferol), is synthesized in the skin from 7-dehydrocholesterol upon exposure to ultraviolet B (UVB) radiation from sunlight. It can also be obtained through diet or supplementation [33,34]. Once produced or ingested, vitamin D_3_ undergoes hydroxylation in the liver, mainly by the cytochrome P450 enzyme CYP2R1, which forms the circulating metabolite 25-hydroxyvitamin D_3_ (25(OH)D_3_) [34]. In the kidneys, a second hydroxylation catalyzed by CYP27BI generates 1,25(OH)_2_D_3_, the most physiologically active form of vitamin D_3_ (Figure 1) [34]. Vit D receptors (VDR) and the activating enzyme 1α-hydroxylase are widely distributed throughout the brain, suggesting roles for Vit D in multiple neurological functions [35,36]. High VDR expression in the hippocampus has prompted research into its potential role in cognitive processes, supported by associations between low Vit D levels and cognitive decline [37]. In developing brains, Vit D regulates neuronal proliferation, differentiation, and maturation [38,39,40,41]. More broadly, Vit D exerts neuroprotective effects by modulating neurotransmission, calcium signaling, and synaptic plasticity, as well as antioxidant responses and neuroimmune regulation [37].

Dysfunction in cortico-basal ganglia dopaminergic pathways is implicated TD pathophysiology, and evidence suggests that Vit D may help maintain dopaminergic system integrity [4,22]. In experimental models of dopaminergic toxicity, the active form of vitamin D_3_ (1,25(OH)_2_D_3_) enhances dopaminergic neuron survival [31]. Vit D may exert neuroprotective effects in developing dopaminergic neurons [42,43] by modulating the RET receptor. RET is a key component of the glial cell line-derived neurotrophic factor (GDNF) signaling pathway, which governs neuronal differentiation and cell survival [44,45,46,47]. Beyond its influence on DA, Vit D may also interact with other neurotransmitter systems relevant to TD. For example, one proposed mechanism involves modulation of the GABAergic system. In a study by Lerner et al., impaired GABA-A receptor binding was observed across several brain regions in individuals with TS, with particularly marked abnormalities in the insula and cerebellum, regions implicated in tic generation [48]. Vit D also regulates neuroinflammatory processes, exerting anti-inflammatory effects through modulation of both cellular and humoral immune responses [49,50]. Low Vit D levels may therefore promote neuroinflammation, a mechanism potentially contributing to TD neuropathology [51]. This relationship is supported by evidence linking low serum Vit D to elevated inflammatory markers in individuals with TD [52].

A growing body of research suggests that serum Vit D levels are lower in patients with TD when compared with healthy controls (Table 1) and that Vit D deficiency may represent a risk factor for neurological disorders, particularly those associated with dopaminergic dysregulation [31,39,53]. A study of children with CTD (*n* = 176; median age of 9 years) and healthy children (*n* = 154) measured 25-hydroxyvitamin D (25[OH]D) levels using high-performance liquid chromatography (HPLC) and tandem mass spectrometry (MS/MS) [52]. Tic status was also measured using the Yale Global Tic Severity Scale (YGTSS). Children with CTD showed significantly lower average 25(OH)D levels (21.7 ng/mL) than healthy children (24.1 ng/mL; *p* = 0.01). These levels were inversely related to motor tic stores on the YGTSS [52]. Vitamin D deficiency was also found to be significantly more common in CTD children (75 ng/mL) than in healthy controls (44 ng/mL) [52]. This finding was replicated by another study that measured serum 25(OH)D levels in children with TD (*n* = 132) and healthy peers (*n* = 144) using HLPC-MS/MS [54]. The researchers then used 25(OH)D levels to categorize each participant’s vitamin D status, which they defined as normal (>30 ng/mL), insufficient (10–30 ng/mL), or deficient (<10 ng/mL) [54]. They found that mean serum 25(OH)D levels were significantly lower (*p* < 0.01) and that vitamin D deficiency or insufficiency rates were significantly higher (*p* < 0.01) in the TD group than in the control group [54]. Another study included children with TD (*n* = 179, 31 females, 148 males, mean age at diagnosis: 8.0 ± 2.7 years old) and without TD (*n* = 189, 35 females, 154 males, mean age at diagnosis: 8.1 ± 2.6 years old). Those with TD had lower mean serum 25(OH)D levels (33 nmol/L) than their healthy peers (84 nmol/L; *p* < 0.001) [55]. The authors also found that 25(OH)D status was significantly associated with the presence of TD, and that among children with TD, 25(OH)D levels were negatively correlated with tic severity scores on the YGTSS [55]. Finally, the rate of 25(OH)D deficiency was significantly higher in the TD group than in the healthy control group [55]. Similar associations have been documented in several independent pediatric cohorts [19,56,57,58,59]. In a larger study, children aged 5–14 years with TD (*n* = 2960, 2355 boys, 560 girls) were compared with age-matched healthy controls (*n* = 2665, 1912 boys, 753 girls), and 25(OH)D levels were measured in both groups [19]. Average levels of 25(OH)D were lower in the TD group (38.47 nmol/L) than in the control group (50.05 nmol/L; *p* < 0.001) [19]. Additionally, 25(OH)D deficiency was significantly more common in those with TD than in those without TD (*p* < 0.001) [19].

Vit D supplementation is increasingly being investigated as a potential adjunctive therapy for TD [60,61]. For example, a rat study examined effects of vitamin D supplementation in animals (*n* = 10) with TS-like symptoms induced by 3,3-Iminodipropionitrile (IDPN) [60]. Administering vitamin D continuously by gavage for 8 weeks at 10 μg/kg/day led to a significant reduction in the tic phenotype over the course of treatment (*p* < 0.001) [60]. Another randomized controlled trial involving 83 children with TDs aged 4–15 years evaluated the comparative efficacy of high-dose (5000 IU/day) and low-dose (1000 IU/day) Vit D supplementation over 3 months [61]. At both dosages, participants showed increased 25(OH)D concentration (*p* < 0.05) and significantly reduced tic severity scores on the YGTSS (*p* < 0.05) [61]. These effects were particularly pronounced in the high-dose group, whose YGTSS scores decreased from 28.85  ±  7.19 at baseline to 18.13  ± 4.30 following treatment (*p* < 0.05). Symptom improvements were accompanied by an increase in 25(OH)D levels from 20.25 ± 7.02 ng/mL to 48.65  ±  10.07 ng/mL (*p* < 0.05) [61]. Moreover, multivariate linear regression analysis revealed a significant negative association between 25(OH) levels and tic severity scores (*p* < 0.05), supporting a dose-dependent relationship between vitamin D status and symptom improvement [61]. In another study comparing 140 controls aged 5 to 14 years with 120 children of the same age with CTD, 36 of whom received Vit D treatment for 3 months, baseline serum 25(OH)D concentrations were significantly lower in CTD participants and negatively correlated with symptom severity [62]. Children with CTD who received vitamin D_3_ supplementation (300 IU/kg/day; maximum 5000 IU/day) showed symptom improvement as their serum 25(OH)D levels approached the target range of 55–70 ng/mL [62]. Following supplementation, children with CTD exhibited normalized serum 25(OH)D levels, which increased from 21 mg/mL at baseline, consistent with vitamin D insufficiency, to 51 ng/mL, falling within the optimal range of 30–90 ng/mL. These changes were accompanied by marked improvements in motor and phonic tics and overall YGTSS scores, without any reported adverse effects [62]. Overall, these findings suggest that Vit D supplementation may constitute a safe and effective adjunctive strategy for managing TD symptoms, particularly in pediatric populations (Table 2).

Caution is warranted when considering Vit D supplementation, as excessive intake may result in toxicity. Because Vit D enhances calcium absorption in the gastrointestinal tract, toxicity is typically characterized by pronounced hypercalcemia, defined as total serum calcium concentrations exceeding 11.1 mg/dL (normal range: 8.4–10.3 mg/dL) [72,73]. Vit D toxicity can also lead to hypercalciuria and markedly elevated serum 25(OH)D concentrations, often exceeding 375 nmol/L (150 ng/mL) [72,73]. Symptoms of hypercalcemia may present as gastrointestinal complaints such as nausea and vomiting. More systemic effects can include dehydration, loss of appetite, increased thirst and urination, kidney stones, neuropsychiatric disturbances, and musculoskeletal pain and weakness [74]. Current guidelines indicate that the tolerable upper intake level for Vit D varies by age, ranging from 25–100 mcg (1000–4000 IU/day) (Table 3) [74].

## 3. Vitamin B6 (Vit B6)

**Figure 2:**
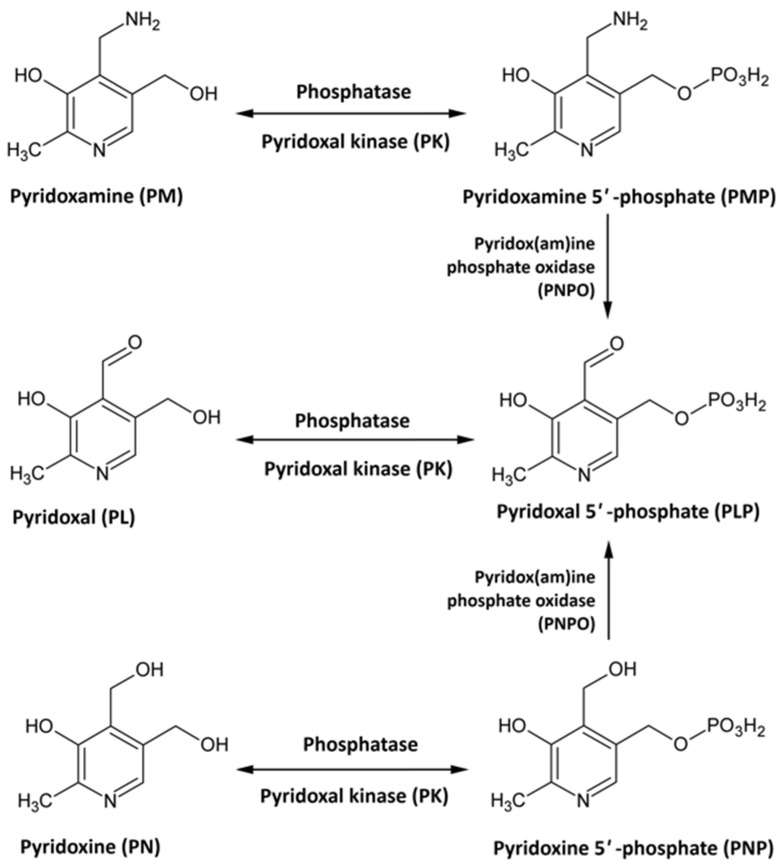
Vitamin B6 metabolism.

Vit B6 is a water-soluble vitamin that functions as a coenzyme in more than 150 enzymatic reactions, primarily involved in the metabolism of amino acids, carbohydrates, and lipids [76,77,78,79]. Beyond its metabolic roles, Vit B6 supports the synthesis of neurotransmitters, heme, and several bioactive metabolites [76,77,78,79]. It also contributes to DNA and RNA synthesis and modulates gene expression [80]. Additionally, Vit B6 maintains chromosomal stability and regulates inflammation, oxidative stress, angiogenesis, and cell cycle progression [81,82,83]. Vit B6 exists in several interconvertible forms, including pyridoxine (PN), pyridoxamine (PM), and pyridoxal (PL) [84,85]. Each vitamer can be phosphorylated by pyridoxal kinase to produce its respective 5′-phosphate derivative. Pyridoxine 5′-phosphate (PNP) and pyridoxamine 5′-phosphate (PMP) are then oxidized by pyridoxine 5′-phosphate oxidase to generate pyridoxal-5′-phosphate (PLP), the biologically active coenzyme form of Vit B6 (Figure 2) [84,85]. Owing to its broad biological functions, particularly in neurotransmitter synthesis, Vit B6 is essential for normal brain function [86,87,88]. PLP acts as a cofactor for glutamate decarboxylase, the enzyme responsible for converting glutamate, the principal excitatory neurotransmitter, into GABA, the main inhibitory neurotransmitter [84,85]. Other neurotransmitter systems also depend on Vit B6, as it is required for the synthesis of serotonin, dopamine, noradrenaline, histamine, glycine, and D-serine [63,79,89,90,91,92,93].

Consequently, Vit B6 deficiency can reduce neurotransmitter levels, potentially contributing to neurological and psychiatric disorders [94]. Multiple lines of evidence link diminished Vit B6 activity to biological abnormalities, including increased sympathetic activation, heightened glucocorticoid sensitivity, and elevated kynurenine levels [63]. Each of these changes can induce neurotoxicity and immune dysregulation [63]. Regarding immune function, Vit B6 deficiency impairs cell-mediated immunity and decreases antibody activity [94]. Deficits in Vit B6 have also been associated with behavioral and motor abnormalities such as hyperirritability, abnormal head movements, and spasmodic motor activity [63]. With respect to its effect on neurotransmission, increasing Vit B6 levels may enhance DA synthesis and restore dopaminergic balance [79,95].

Accordingly, several studies suggest that Vit B6 supplementation may support the treatment of neurological disorders including TS and other TD. A double-blind randomized controlled trial reported that daily Vit B6 at 2 mg/kg reduced TS symptoms in children aged 7–14 years, reflected in lower YGTSS scores and marked reductions in both motor and phonic tics [63]. Another open-label randomized trial of children aged 4–17 years with TS or CTD (*n* = 34, 30 boys and 4 girls) found decreased tic and anxiety symptoms following Vit B6-based nutritional supplementation [64]. One group of participants (*n* = 17) was randomly assigned to receive Vit B6 at 2.8 mg/day for two months, while the other group (*n* = 17) did not receive supplementation. In the Vit B6 group, mean YGTSS scores decreased from 20.35 (±5.8) to 11.5 (±6.1) after treatment, with an average score reduction of 8.85 (43.5%) [64]. This represented a significantly greater improvement in tic severity over the course of the study compared to the control group [64]. Similarly, in a study by Garcia-López et al., supplementation of Vit B6 along with Mg correlated with lower YGTSS scores during symptomatic exacerbations in children aged 7 to 14 years over 90 days of treatment [65]. The total effect on the YGTSS was a decrease from 58.1 at baseline to 12.9 post-treatment, and total tic scores lowered from 26.7 to 12.9, both changes being statistically significant [65]. Collectively, these findings indicate that Vit B6 supplementation may represent a safe, viable adjunctive approach for managing TD symptoms (Table 2).

Safety is an important consideration for Vit B6 supplementation. Overconsumption of Vit B6 can cause neuropathy, which may lead to impaired motor control [96,97]. To minimize the risk of toxicity, the tolerable upper intake level of Vit B6 for adults is set to 100 mg per day (Table 3) [97].

## 4. Vitamin A (Vit A)

Vit A is a vital micronutrient required for the normal functioning of several body systems, including the central nervous system [60,95]. Its active metabolite, retinoic acid (RA), is essential for neuronal developmental processes such as differentiation and maturation [52,60,98]. RA also exhibits antioxidant properties and regulates inflammatory signaling [99,100,101]. In addition, Vit A influences gene expression through nuclear retinoic acid receptor (RAR) complexes [102,103]. In the brain, Vit A supports neuroplasticity, with animal studies showing that Vit A deficiency impairs hippocampal plasticity through mechanisms involving RA nuclear receptor-α (RARα) [104,105,106]. RARs are abundantly expressed in striatal neurons, where RA signaling promotes neurogenesis and striatal differentiation [107,108]. By modulating gene transcription, RA can affect DA pathways, suggesting a potential role for Vit A in TD pathogenesis, which is consistently linked to dopaminergic dysfunction [105,109,110].

Several clinical studies corroborate this association. In one study comparing children with CTD (*n* = 176, median age of 9 years) and healthy controls (*n* = 154, median age of 9 years), Vit A deficiency and combined micronutrient insufficiency were significantly more prevalent in the CTD group [52]. Vit A status was determined by circulating retinol levels, which were measured by HPLC and MS/MS [52]. The CTD group, whose tic status was confirmed with the YGTSS, had a mean serum retinol level of 1.09 μmol/L, while the control group had a significantly higher mean of 1.23 μmol/L (*p* = 0.001) [52]. Another study involving 198 children with TD and 50 controls found markedly lower Vit A levels and higher deficiency rates among affected participants, with the lowest concentrations observed in those with TS compared to CTD or TTD (*p* < 0.05) [56]. A larger study of 245 children with TD and 63 controls further revealed an inverse correlation between serum Vit A concentrations and tic severity (*p* < 0.01) [66]. Collectively, these findings suggest that insufficient Vit A status may contribute to TD pathogenesis and suggest possible benefits from Vit A supplementation. In one intervention study, 50 children with TD and 50 neurotypical peers received Vit A drops combined with five-dimensional lysine granules, which resulted in short-term tic reduction [111]. This outcome highlights the possibility of a potential therapeutic role for Vit A supplementation in managing TD symptoms (Table 2).

Excessive Vit A intake, referred to as hypervitaminosis A, can result in significant hepatotoxicity [112]. Toxicity may occur acutely, following a very high single dose, or chronically, arising from prolonged overconsumption. Clinical manifestations include liver damage, as well as nausea and bone pain [113]. Mechanistically, Vit A toxicity contributes to oxidative stress, inflammation, and progressive liver damage which may advance from steatosis to fibrosis and ultimately cirrhosis [113,114]. The tolerable upper intake level for adults has been established as 3000 retinol activity equivalents (RAE) per day, corresponding to 3000 mcg or 9000 IU (Table 3) [115].

## 5. Iron

Iron is a trace element that serves as a cofactor for numerous enzymes involved in hydroxylation, oxidation, and peroxidation reactions [70,116]. Consequently, it is indispensable for several biological processes, including oxygen transport and mitochondrial energy production [117,118]. Iron also plays a central role in neurotransmitter systems, particularly for monoamines such as dopamine, norepinephrine, epinephrine, and serotonin. These systems regulate emotion, attention, reward processing, and motor control [119]. Iron is essential for neurotransmitter synthesis, transport, and metabolism [120], particularly within dopaminergic circuits, where it facilitates DA synthesis, receptor development, and transporter function [121,122]. Additionally, iron helps maintain glutamate and γ-aminobutyric acid (GABA) equilibrium, thereby sustaining the excitatory–inhibitory balance critical for normal neural communication [123,124,125,126]. Iron deficiency has been associated with alterations in GABA metabolism, including elevated GABA concentrations in the hippocampus, striatum, and globus pallidus, as well as changes in GABA receptor binding at synaptic membranes [127,128,129,130]. In addition to neurotransmitter metabolism, iron supports neuronal development by promoting dendritic and synaptic maturation, as well as white matter myelination [131,132,133].

Low iron levels have been associated with central nervous system dysfunction across several neuropsychiatric disorders, including TS [134,135,136,137]. A neuroimaging study reported that, relative to matched controls (*n* = 40), individuals with TS (*n* = 25) exhibited significantly reduced subcortical magnetic susceptibility, indicative of lower iron content, in regions such as the caudate, pallidum, subthalamic nucleus, thalamus, red nucleus, and substantia nigra [116]. These alterations were accompanied by dopaminergic dysregulation, including decreased availability of dopamine D1 receptors in the dorsal striatum, which positively correlated with tic severity [116]. Together, these findings suggest that disturbances in multiple dopaminergic components, particularly including synaptic dopamine release and D1 receptor binding, may contribute to TS neuropathology [116]. The co-occurrence of these abnormalities with disrupted iron homeostasis further implicates iron dysregulation as one factor in TS pathogenesis.

Other studies indicate a possible association between reduced iron levels and structural brain alterations that may contribute to TD development [28,67,68]. Evidence suggests that inadequate iron availability could lead to hypoplasia, or incomplete maturation, of the caudate putamen, potentially increasing susceptibility to tic formation [28]. Lower iron concentrations may also be linked to decreased cortical volumes, resulting in diminished inhibitory control of motor output [67]. One study measured serum iron in 41 children and adults with TS and 32 controls. Ferritin, a key iron-storage protein, was measured in 63 individuals with TS and 44 controls [67]. Consistent with prior research, the investigators observed lower serum iron and ferritin levels in participants with TS, though values remained within physiological norms and were not significantly associated with symptom severity [67]. Mean serum ferritin in the TS group was 55.0 ng/mL (SD = 54.3), significantly lower than 72.2 ng/mL (SD = 72.8) in the control group (t = 2.28, df = 106, *p* = 0.03) [67]. As with ferritin, serum iron levels were within the normal range in both groups (60–180 μg/dL in males or 50–170 μg/dL in females). Still, mean serum iron was lower in the TS group at 81.6 μg/dL (SD = 24.8) than in the control group at 90.6 μg/dL (SD = 24.8), and this difference was statistically significant (t = 2.38, df = 72, *p* = 0.02) [67]. Neuroimaging analyses further explored mechanisms linking iron availability to TS pathology. Magnetic resonance imaging (MRI) revealed contrasting correlations between ferritin levels and brain morphology in the TS and control groups: in the TS group, ferritin levels positively correlated with caudate volume, whereas in the controls, the relationship was inverse [67]. Across both cohorts, ferritin levels positively correlated with cortical volumes in the sensorimotor, midtemporal, and subgenual cortices [67], further supporting the idea that iron availability can influence brain structure. A large retrospective study comparing medical records from children with TD (*n* = 1204) and controls (*n* = 1220) found that those with TD had lower whole blood iron levels (*p* < 0.01) [68]. The average Fe level in the TD group was 8.47 ± 0.86 mmol/L, compared to 8.80 ± 0.94 mmol/L in the control group [68]. Another study conducted in a pediatric neurology clinic assessed iron store balance by measuring serum ferritin in drug-naïve children receiving a first-time diagnosis of a TD (*n* = 47, 32 boys and 15 girls, aged 3.17–8.66 years), compared to age- and sex- matched children seen in the same clinic for headaches (*n* = 100, 62 boys and 38 girls, aged 3.17–8.66 years) [69]. Mean serum ferritin levels were 32% lower in children with TD than in their peers with headaches (*p* = 0.01) [69].

Building on evidence linking iron balance to TD, multiple studies have demonstrated significant correlations between reduced iron availability and increased tic severity. A case–control study of 54 children with TD and 54 matched healthy controls reported significantly lower serum iron and ferritin levels in the TD group, alongside strong negative correlations between these measures and YGTSS scores [59]. This trend extended to clinical subtypes of CTD and TS, where lower iron and ferritin concentrations corresponded with more severe tic manifestations [59]. In one study, children with TD (*n* = 57) who exhibited serum ferritin levels below 50 ng/mL (*n* = 37) displayed higher tic severity and greater life impact scores than those maintaining normal ferritin levels (*n* = 20) [70]. Within 12 months of the initial measurement, 26 patients were seen for follow-up, including 12 who were iron-deficient and 14 who were iron-sufficient at baseline [70]. Iron-deficient children who received iron supplementation (*n* = 5) showed an improvement in tic severity scores from 2.70 to 1.90 after 12 months, whereas those who did not receive supplementation (*n* = 7) saw an increase from 2.36 to 2.70 [70]. Similar effects were seen in the iron-sufficient group, though less pronounced. Iron-sufficient children supplemented with iron (*n* = 10) showed a reduction in tic severity scores from 2.40 to 1.95, while children who were not supplemented (*n* = 4) showed no change, remaining at an average score of 2.88 upon follow-up [70]. This emerging evidence base suggests that iron supplementation may be a promising adjunctive therapeutic approach for TD management. Although further longitudinal trials are warranted to confirm efficacy and safety, emerging evidence provides encouraging support for the role of iron supplementation as an adjunctive intervention in TD treatment (Table 2).

Toxic amounts of iron consumption can lead to various gastrointestinal issues, including nausea, vomiting, diarrhea, constipation, and gastric upset [138]. At very high doses, more severe effects may be seen, including corrosive necrosis of the intestine or multisystem organ failure, which can be fatal [139]. Excess iron accumulates in the liver, heart, and endocrine glands, where it promotes the production of reactive oxygen species, leading to oxidative stress, cell damage, and organ dysfunction [140]. Toxicity can occur due to high individual doses or from gradual toxic accumulation. The latter is particularly of concern in patients who receive chronic transfusions for sickle cell disease, thalassemia, or hematologic malignancies [141]. To minimize the risk of toxicity, the tolerable upper intake level for iron is set at 45 mg per day in adults. For infants, children, and adolescents, the upper intake level varies by age, ranging from 40–45 mg per day (Table 3) [139].

## 6. Magnesium (Mg)

Mg is an essential mineral that acts as a cofactor in more than 600 biochemical reactions, making it indispensable for maintaining physiological stability and overall health [142,143,144,145,146,147]. One of Mg’s primary functions is regulating intracellular calcium and potassium, two electrolytes critical for neural excitability and synaptic signaling [148]. Within the nervous system, Mg modulates the body’s stress response, partly by influencing the hypothalamic–pituitary–adrenal (HPA) axis [149]. It decreases adrenocorticotropic hormone (ACTH) secretion from the pituitary and cortisol release from the adrenal glands, thereby mitigating anxiety [150,151]. Beyond its endocrine effects, Mg attenuates presynaptic glutamate release and glutamatergic hyperactivity, processes that are implicated in fear and panic responses [149,152,153,154,155].

Given its extensive neuro-modulatory functions, low Mg levels can contribute to psychiatric manifestations such as anxiety and depression, as well as neurological manifestations including headaches, seizures, muscle spasms, and tics [156,157,158]. Studies indicate that Mg deficiency induces neuromuscular hyperexcitability, often accompanied by convulsions and involuntary movements such as chorea and athetosis [159]. Mg insufficiency has also been linked to increased DA release, altered serotonin receptor modulation, and heightened defensive reactivity [28]. In the context of TD, Mg deficiency appears more prevalent among children with TS than among healthy peers [159]. However, a large retrospective analysis of medical records from 1204 children with TD and 1220 controls found no significant differences in whole-blood Mg concentrations between groups [68]. Children with TD had a mean Mg level of 1.58 ± 0.19 mmol/L, whereas controls had a mean Mg level of 1.57 ± 0.17 mmol/L (*p* = 0.318) [68].

Despite these inconsistencies, several studies suggest that Mg supplementation may alleviate tic severity. A randomized, double-blind controlled trial in children aged 7–14 years experiencing TS exacerbations found that daily Mg administration (0.5 mEq/kg) significantly reduced motor and phonic tics and improved overall impairment on the YGTSS [63]. In this study, the Mg group showed a 50% improvement from baseline, with a mean total tic count of 12.9 (SD = 11.20), compared with 26.7 (SD = 7.38) in the control group [63]. The authors presented a power calculation indicating that mean final total tic scores of 13 (SD = 10) for the treatment group and 23 (SD = 10) for the control group yielded a statistical power of 0.8 and a significance level of 0.05 [63]. Another study involving children aged 7–14 with TS reported progressive tic reduction over 90 days following oral administration of Mg and Vit B6, with symptom relief observed during clinical exacerbations [65]. Total tic scores lowered from 26.7 at baseline to 12.9 after 90 days of treatment, and the total effect on the YGTSS was a decrease from 58.1 to 18.8 [65]. Collectively, these findings highlight the therapeutic potential of Mg supplementation as an adjunctive intervention for TS and other TD, although further trials are warranted to establish efficacy and optimal dosing parameters (Table 2).

Excessive intake of magnesium can result in hypermagnesemia, which may manifest as gastrointestinal symptoms including diarrhea, nausea, and abdominal cramping [160]. At extremely high intakes, magnesium toxicity can cause serious cardiovascular effects, including arrhythmias and, in severe cases, cardiac arrest [161]. Individuals with impaired renal function or kidney failure are particularly at risk of toxicity, as their ability to remove excess magnesium is reduced or absent [161]. The tolerable upper intake level of magnesium for adults is set at 350 mg per day (Table 3) [162].

## 7. Zinc (Zn)

Zn is an essential trace element that acts as a cofactor for more than 300 enzymes involved in carbohydrates, protein, fatty acid, and nucleic acid metabolism [163,164,165,166]. These processes are vital for central nervous system development and maintenance [167,168]. In addition to neurogenesis, Zn exerts protective effects throughout the body by supporting immune function and antioxidant defenses [169,170]. At the molecular level, Zn acts as a cofactor for zinc-finger proteins, which contribute to neuronal metabolism by regulating gene expression and enzymatic activation [169]. Proper Zn homeostasis is essential for brain health, as both deficiency and excess can induce neural dysfunction [171]. Zn deficiency has been linked to oxidative stress and cognitive impairment, whereas accumulation beyond physiological levels can trigger neurotoxicity and neuronal apoptosis [169,171]. Within the brain, Zn contributes to melatonin synthesis in the pineal gland, thereby modulating DA activity [172]. This is noteworthy since dopaminergic dysregulation remains a defining feature of TD pathophysiology, and downregulating DA signaling is considered a central therapeutic strategy. Zn may also influence TD symptoms by modulating GABA neurotransmission, which exerts inhibitory effects on tic generation. Accordingly, Zn deficiency, which has been associated with impaired GABA synthesis in rat studies, may exacerbate tics [173]. Several studies have investigated Zn status in TD populations [68,174]. A large retrospective review of medical records from 1204 children with TD and 1220 healthy controls revealed significantly lower whole-blood Zn concentrations in the TD group (78.90 ± 11.50 μmol/L) compared to controls (83.90 ± 12.10 μmol/L; *p* < 0.01) [68]. Similarly, an outpatient study involving 161 children with TD and 178 healthy peers reported lower serum Zn levels among those with TD [71]. Collectively, these studies support an association between Zn deficiency and TD. However, evidence for the therapeutic efficacy of Zn supplementation remains inconclusive, underscoring the need for controlled interventional research to clarify its role in symptom modulation (Table 2).

While Zn is essential in trace amounts, excessive intake can result in toxicity, affecting the nervous, gastrointestinal, and respiratory systems [175]. Overconsumption of Zn may also induce relative copper deficiency, which can cause neurological symptoms such as numbness and weakness in the limbs [176]. To reduce the risk of toxicity, the European Food Safety Authority (ESFA) recommends a tolerable upper intake level of 25 mg per day for adults, whereas the U.S. Food and Drug Administration (FDA) sets the upper intake level at 40 mg per day (Table 3) [177].

## 8. Copper (Cu)

Cu is an essential trace element that plays a pivotal role in brain function. It supports enzymatic processes involved in DA metabolism, norepinephrine synthesis, catecholamine degradation, and cellular antioxidant defense [178,179,180]. Cu acts as a cofactor for multiple cuproenzymes, including cytochrome c oxidase, dopamine β-hydroxylase, amine oxidase, tyrosinase, and Cu-dependent superoxide dismutase [181,182]. These enzymes facilitate oxidation, hydroxylation, and disproportionation reactions vital to cellular energy metabolism [181,182]. Through these enzymatic functions, Cu contributes to mitochondrial respiration, oxidative stress regulation, and the biosynthesis of pigments, connective tissue, and neurotransmitters [183]. Dysregulation of Cu homeostasis has been implicated in several neurological disorders, particularly those affecting motor control. Elevated Cu concentrations may promote neurotoxicity through the formation of Cu–dopamine complexes, which oxidize DA and generate reactive oxygen species, ultimately causing dopaminergic neuronal injury [184,185]. This mechanism provides a plausible link between excess Cu and disorders characterized by dopaminergic dysregulation, including TD [184,185]. Although evidence on Cu involvement in TD remains limited, some studies suggest a potential association. In the same large retrospective study of 1204 children with TD and 1220 controls that identified lower Zn levels in the TD group, children with TD also exhibited significantly reduced whole blood Cu concentrations compared with healthy peers [68]. Cu levels were 17.80 ± 3.28 μmol/L in the TD group and 18.50 ± 3.54 μmol/L in the control group (*p* < 0.01) [68]. At present, there is insufficient evidence to support Cu supplementation as a therapeutic intervention for TD, highlighting the need for further mechanistic and interventional research (Table 2).

Cu toxicity resulting from excessive consumption may cause liver damage and gastrointestinal symptoms, including abdominal pain, nausea, vomiting, and diarrhea [186,187]. Maintaining an appropriate balance between Cu and Zn is important for overall health [188]. Normal serum levels are 10–25 µmol/L for copper and 12–15 µmol/L for zinc [189,190]. Disturbances in the copper-to-zinc ratio have been associated with serious diseases and health problems, highlighting the need for careful consideration when supplementing with either trace element [190]. The tolerable upper intake level for Cu in adults is set at 10,000 mcg per day (Table 3) [190].

## 9. Non-Nutritional Strategies

The management of tic disorders is complex and can generally be divided into three main approaches: (1) pharmacological therapy, (2) behavioral therapy, and (3) neurostimulation [191,192].

Pharmacologic treatment options include alpha-2-agonists, anticonvulsants, dopamine receptor antagonists, dopamine depletors, and muscle relaxants [192,193]. Other options include cannabis and traditional Chinese herbal therapies [192,193]. When first-line options are insufficient, second-line strategies often involve the use of antipsychotics such as fluphenazine, aripiprazole, risperidone, and ziprasidone [191]. While generally effective, these medications carry the risk of inducing side effects such as metabolic syndrome and tardive dyskinesia [191]. Only three antipsychotic agents (haloperidol, pimozide, and aripiprazole) have been approved by the FDA for managing TD symptoms [194]. Of these, aripiprazole belongs to the class of atypical antipsychotics, which are more selective dopamine D2 receptor blockers, although they may also influence serotonin signaling [195]. Other atypical antipsychotics include risperidone, clozapine, olanzapine, and quetiapine [195].

Non-pharmacologic strategies provide alternatives for patients who do not respond to drug therapy or who wish to avoid systemic side effects [196]. The American Academy of Neurology (AAN) recognizes behavioral therapy and deep brain stimulation (DBS) as the two primary non-pharmacologic interventions [194]. Behavioral therapy is widely considered the most effective psychotherapeutic approach for tic disorders [197,198]. This is often delivered as Comprehensive Behavioral Intervention for Tics (CBIT), which integrates three previously separate interventions: habit reversal training, relaxation therapy, and awareness training [191]. CBIT has received a “high confidence” recommendation in the AAN’s clinical practice guidelines and is regarded as a non-invasive first-line treatment for TS [196]. DBS is a relatively new intervention in which electrodes are surgically implanted into targeted brain regions, enabling modulation of neural activity through electrical stimulation [194,197]. In tic disorders, this targeted modulation may help normalize dysfunctional neural circuits involved in generating tics.

## 10. Conclusions

TDs are neurodevelopmental conditions characterized by persistent motor and/or vocal tics, with diagnostic categories including TS, CTD, and TTD. This review examined the association between TD, particularly TS and CTD, and serum concentrations of several key micronutrients (Vit D, Vit B6, Vit A, iron, Mg, Zn, and Cu). These micronutrients play critical neurobiological roles, and their insufficiencies may contribute to TD pathophysiology by disrupting neurotransmitter regulation, neurodevelopmental processes, and cellular redox homeostasis. Accumulating evidence suggests association of deficiencies in these micronutrients with both the occurrence and severity of TDs. Preliminary clinical studies indicate that supplementation with specific nutrients including Vit D, Vit B6, Vit A, iron, Mg, Zn, and Cu may alleviate tic symptoms, although some findings are variable across studies. Further neurochemical, genetic, and longitudinal research is warranted to clarify the mechanisms underlying these associations and to evaluate the efficacy of nutrient-based interventions as adjunctive strategies for TD management and prevention.

## Figures and Tables

**Table 1:** Comparison of serum 25-hydroxyvitamin D levels between TD group and healthy controls.

Study	Sample Size (TD/HC)	Design Type	Exposure Assessment	Vitamin D Deficiency Threshold	Serum 25-Hydroxyvitamin D Level		
					TD	HC	*p* Value
Wang et al., 2022 [19]	2960/2665 TD (*n* = 2960, 2355 M, 560 F), HC (*n* = 2665, 1912 M, 753 F), aged 5–14 years.	Retrospective case–control	Serum 25(OH)D (nmol/L)	≤37.5 nmol/L = deficient; 37.5–50 nmol/L = insufficient; ≥50 nmol/L = sufficient	38.47 (23.56–58.89) nmol/L	50.05 (34.47–65.80) nmol/L	*p* < 0.001
Wang et al., 2024 [52]	176/154 CTD (*n* = 176, 131 M, 45 F), HC (*n* = 154), median age of 9 years.	Case–control	Serum 25(OH)D (ng/mL)	<20 ng/mL = deficient; 21–29 ng/mL = insufficient; 30–90 ng/mL = optimal	21.7 (16.6–27.8) ng/mL	24.1 ng/mL (19.4–28.8) ng/mL	*p* = 0.01.
Li et al., 2017 [54]	132/144 TD (*n* = 132, 108 M, 24 F), average age of 8.4 years. HC (*n* = 144, 117 M, 27 F), average age of 8.3 years.	Case–control	Serum 25(OH)D (ng/mL)	<10 ng/mL = deficient; 10–30 ng/mL = insufficient; ≥30 = optimal	23 ± 9 ng/mL	32 ± 8 ng/mL	*p* < 0.001
Li et al., 2018 [55]	179/189 TD (*n* = 179, 148 M, 31 F, mean age: 8.0 years. HC (*n* = 189, 35 F, 154 M, mean age: 8.1 years.	Case–control	Serum 25(OH)D (ng/mL)	<10 ng/mL = deficient; 10–30 ng/mL = insufficient; ≥30 = optimal	22.9 ± 7.5 ng/mL	28.9 ± 8.3 ng/mL	*p* < 0.001

Abbreviations: M, Male/boys; F, Female/girls; 25(OH)D, 25-hydroxyvitamin D.

**Table 2:** Summary of studies that support the role of micronutrients in tic alleviation.

Study	Sample Size (TD/HC), Age	Design Type	Outcome
Vitamin D			
Wang et al., 2022 [19]	2960/2665, 5–14 years	Retrospective case–control	Serum 25-hydroxyvitamin D Levels were lower in the TD group (38.47 nmol/L) than in healthy control (50.05 nmol/L).
Wang et al., 2024 [52]	176/154, 9 years	Case–control	Serum 25-hydroxyvitamin D Levels were lower in the TD group (21.7 ng/mL) than in healthy control (24.1 ng/mL).
Li et al., 2017 [54]	132/144, 8.4 years	Case–control	Serum 25-hydroxyvitamin D Levels were lower in the TD group (23 ± 9 ng/mL) than in healthy control (32 ± 8 ng/mL).
Li et al., 2018 [55]	179/189, 8.0 years	Case–control	Serum 25-hydroxyvitamin D Levels were lower in the TD group (22.9 ng/mL) than in healthy control (28.9 ng/mL).
Mohamed et al., 2025 [61]	83, 4–15 years	Randomized controlled trial	Participants received high-dose (5000 IU/day) and low-dose (1000 IU/day) Vit D supplementation over 3 months. At both dosages, participants showed increased 25(OH)D concentration and significantly reduced tic severity scores on the YGTSS.
Li et al., 2019 [62]	36, 5 to 14 years		Participants who received Vit D supplementation (300 IU/kg/day; maximum 5000 IU/day) for 3 months showed symptom improvement.
Vitamin B6			
Garcia-Lopez et al., 2009 [63]	19/19, 7–14 years	Randomized controlled trial	Daily Vit B6 supplementation (2 mg/kg) reduced TS symptoms at 90 days.
Rizzo et al., 2022 [64]	17/17, 4–17 years	Randomized trial	Individuals received B6 supplementation 2.8 mg/day for two months, and their mean YGTSS scores decreased from 20.35 (±5.8) to 11.5 (±6.1).
García-López et al., 2008 [65]	Children with TS, 7 to 14 years	Placebo treatment	Children received B6 supplementation, along with Mg. Total tics scores decreased from 26.7 (at 0 days) to 12.9 (at 90 days) and the total effect on the YGTSS was a reduction from 58.1 to 18.8.
Vitamin A			
Wang et al., 2024 [52]	176/154, 9 years	Case–control	Serum retinol levels were low in the CTD group. The CTD group had a mean serum retinol level of 1.09 μmol/L, while the control group had a mean serum retinol level of 1.23 μmol/L.
Wang et al., 2022 [56]	198/50		Lowest serum Vit A concentrations were observed in TS compared to CTD or TTD (*p* < 0.05).
Hou et al., 2020 [66]	245/63		An inverse correlation was found between serum Vit A concentrations and tic severity (*p* < 0.01).
Iron			
Gorman et al., 2006 [67]	41/32	Comparative study	Mean serum iron was significantly lower in the TS group at 81.6 μg/dL than in the control group at 90.6 μg/dL, and this difference was statistically significant (t = 2.38, df = 72, *p* = 0.02).
Qian et al., 2019 [68]	1204/1220	Retrospective study	Those with TD had lower average whole blood iron levels (8.47 ± 0.86 mmol/L) than controls (8.80 ± 0.94 mmol/L).
Avrahami et al., 2017 [69]	47/100, 3–8 years	Cross-sectional	Mean serum ferritin levels were 32% lower in children with TD than in controls.
Wang et al., 2022 [59]	54/54		Lower serum iron and ferritin levels were found in the TD group, alongside strong negative correlations between these measures and YGTSS scores.
Ghosh et al., 2017 [70]	Children with TD (*n* = 57)		Iron-deficient children who received iron supplementation (*n* = 5) showed an improvement in tic severity scores from 2.70 to 1.90 upon 12 months, whereas those who did not receive supplementation (*n* = 7) saw an increase from 2.36 to 2.70.
Mg			
Garcia-Lopez et al., 2009 [63]	19/19, 7–14 years	Randomized controlled trial	Daily Mg administration (0.5 mEq/kg) significantly reduced motor and phonic tics and improved overall impairment on the YGTSS.
Qian et al., 2019 [68]	1204/1220	Retrospective study	Children with TD had a mean Mg level of 1.58 ± 0.19 mmol/L, whereas controls had a mean Mg level of 1.57 ± 0.17 mmol/L (*p* = 0.318).
García-López et al., 2008 [65]	Children with TS, 7 to 14 years	Placebo treatment	Children received Mg supplementation, along with Vit B6. Total tics scores decreased from 26.7 (at 0 days) to 12.9 (at 90 days) and the total effect on the YGTSS was a reduction from 58.1 to 18.8.
Zn			
Qian et al., 2019 [68]	1204/1220	Retrospective study	Zn levels were lower in children with TD (78.90 ± 11.50 μmol/L) than in controls (83.90 ± 12.10 μmol/L).
Luo et al., 2023 [71]	161/178		Lower serum Zn levels were reported among those with TD.
Cu			
Qian et al., 2019 [68]	1204/1220	Retrospective study	Cu levels were significantly lower in the TD group (17.80 ± 3.28 μmol/L) than in the control group (18.50 ± 3.54 μmol/L).

**Table 3:** Tolerable upper intake levels (ULs) for vitamins D, A, B6, magnesium, iron, zinc, and copper.

Age	Vitamin D	Vitamin A	Vitamin B6	Magnesium	Iron	Zinc	Copper
0–6 months	25 mcg (1000 IU)	600 mcg	NPE	NPE	40 mg	4 mg	NPE
7–12 months	38 mcg (1500 IU)	600 mcg	NPE	NPE	40 mg	5 mg	NPE
1–3 years	63 mcg (2500 IU)	600 mcg	30 mg	65 mg	40 mg	7 mg	1000 mcg
4–8 years	75 mcg (3000 IU)	900 mcg	40 mg	110 mg	40 mg	12 mg	3000 mcg
9–13 years	100 mcg (4000 IU)	1700 mcg	60 mg	350 mg	40 mg	23 mg	5000 mcg
14–18 years	100 mcg (4000 IU)	2800 mcg	80 mg	350 mg	45 mg	34 mg	8000 mcg
19+ years	100 mcg (4000 IU)	3000 mcg	100 mg	350 mg	45 mg	40 mg	10,000 mcg

Abbreviations: NPE, Not possible to establish; IU, International Units; mcg, Micrograms; mg, milligrams. Source: National Institutes of Health (NIH), Office of Dietary Supplements (ODS) [75].

## Data Availability

No new data were created or analyzed in this study. Data sharing is not applicable to this article.

## Abbreviations

The following abbreviations are used in this manuscript:

TS	Tourette syndrome
TD	Tic disorder
TTD	Transient tic disorder
CTD	Chronic motor or vocal tic disorder
OCD	Obsessive–compulsive disorder
ASD	Autism spectrum disorder
ADHD	Attention-deficit/hyperactivity disorder
CSTC	Cortico-striato-thalamo-cortical
DA	Dopamine
GABA	γ-aminobutyric acid
Vit D	Vitamin D
VDR	Vitamin D receptor
UVB	Ultraviolet B
GDNF	Glial cell line-derived neurotrophic factor
YGTSS	Yale Global Tic Severity Scale
HPLC	High-performance Liquid Chromatography
MS/MS	Tandem Mass Spectrometry
HC	Healthy Controls
IU	International Units
Vit B6	Vitamin B6
PLP	Pyridoxal-5′-phosphate
PN	Pyridoxine
PM	Pyridoxamine
PL	Pyridoxal
PNP	Pyridoxine 5′-phosphate
PMP	Pyridoxamine 5′-phosphate
RA	Retinoic acid
RAR	Retinoic acid receptor
UL	Upper intake level
HPA	Hypothalamic–pituitary–adrenal
ACTH	Adrenocorticotropic hormone
Mg	Magnesium
Zn	Zinc
Cu	Copper
MRI	Magnetic resonance imaging
FDA	Food and Drug Administration
AAN	American Academy of Neurology
DBS	deep brain stimulation
CBIT	Comprehensive Behavioral Intervention
